# Chinese and white Canadian satisfaction and compliance with physicians

**DOI:** 10.1186/1471-2296-8-11

**Published:** 2007-03-21

**Authors:** Richard Liu, Lawrence So, Hude Quan

**Affiliations:** 1 Faculty of Medicine and Dentistry, University of Alberta, Canada; 2 Department of Community Health Sciences, University of Calgary, Canada; 3 The Center for Health and Policy Studies, University of Calgary, Calgary, Alberta, Canada

## Abstract

**Background:**

Patient satisfaction has become an important indicator of primary care and healthcare system performance. Ethnic disparities in patient satisfaction and compliance with physician care have been studied in several countries. However, this issue has not received significant attention in Canada. The unique characteristics of the Canadian healthcare system and ethnic population make it worthwhile to examine this issue in this population. Therefore, we conducted a survey among Chinese and Whites in a Canadian city to determine their reported satisfaction, and perceptions of physicians.

**Methods:**

The survey was conducted in English, Mandarin and Cantonese in 2005 among Chinese and White Canadians, 18 years of age or older, who had visited at least one physician in Canada.

**Results:**

We analyzed 746 Chinese and 711 Whites in the general practitioner (GP) visit group and 485 Chinese and 637 Whites in the specialist visit group. A lower proportion of Chinese compared to Whites reported that they were very satisfied or satisfied with GP (73.7% vs. 92.8%) and specialist care (75.5% vs. 85.6%) and the differences between the two groups remained after adjustment for demographic variables and chronic conditions (risk adjusted OR: 0.70, 95%CI: 0.42–1.18 for the GP visit group and OR: 0.77, 95%CI: 0.48–1.23 for the specialist visit group). A similar proportion of Chinese and Whites reported that they always followed a physician's advice (59.4% vs. 59.6% for the GP visit group and 67.2% vs. 62.8% for the specialist visit group). Non-English speaking Chinese and recent arrivals in Canada were less likely to be satisfied with GPs than Chinese born in Canada [risk adjusted OR: 0.5, 95%CI: 0.3–0.9, 0.2 and 0.1–0.7, respectively].

**Conclusion:**

Chinese Canadians reported lower satisfaction with physicians and perceived physicians slightly more negatively than White Canadians. Particularly, Chinese with limited English and short length of stay in Canada were less satisfied than Canadian born Chinese.

## Background

Patient satisfaction has become an important indicator of quality of primary care and healthcare system performance [1-3]. Low patient satisfaction is associated with lower trust in caregivers and greater chance of physician change – resulting in less continuity of care [4]. Not surprisingly, low patient satisfaction is also correlated to greater numbers of patient complaints and malpractice lawsuits [5,6]. Hence, the subject of patient satisfaction and how it benefits both patients and physicians has been widely studied.

It has been observed that various patient-physician interaction styles have had different effects on patient satisfaction [7,8]. Moreover, complaints in hospital settings are twice as likely to be related to communication problems as opposed to problems with medical skill [9]. Patient-physician belief congruence and high patient involvement in care have also been associated with higher trust and satisfaction [10-12]. These factors are all modifiable by physicians or patients, whereby, physicians should adapt to individual patient needs and patients should choose physicians that can adequately fill these needs.

Ethnic disparities in patient satisfaction have been examined in the United States, Australia, and the United Kingdom [13-18]. These studies have shown that Blacks, Hispanics, and Asians tend to rate their healthcare experiences less positively than their White counterparts. Among Asians specifically, lower satisfaction and trust have been consistently found [16,19-21]. Factors underlying these differences include education, socioeconomic status, physical and emotional health, health insurance coverage, healthcare costs, conflicting cultural views on healthcare, and communication difficulties.

Little is known about ethnic Canadians' satisfaction and compliance with physicians and previous study findings generated from other countries cannot be automatically generalized to the Canadian population for two major reasons. Firstly, the ethnic population in Canada differs noticeably from that in the United States and United Kingdom in terms of immigration history and ethnic composition. A majority of Canadian immigrants were selected based on their health status, education and technical skills in contrast to descendents of immigrants through labor forces in both the United States and the United Kingdom and slavery in the United States. Secondly, in the United States, insurance status and socioeconomic differences explain a significant part of the disparities in access to and use of healthcare services by ethnic minorities [22-24]. In Canada, where a universal healthcare system allows equal access to healthcare services, studies have shown that utilization of general practitioners (GPs) and specialists is similar in ethnic minorities and Whites [25]. For these reasons, there is a need for investigation into the healthcare experiences of Canadian ethnic minorities. In particular, the differences in perceptions of physicians by ethnic minorities and Whites and the reasons behind these differences need to be examined.

We conducted a telephone survey among Chinese and Whites in a large Canadian city to determine their reported satisfaction, perceptions of their GPs and specialists, and factors associated with these measurements among Chinese. Chinese Canadians were chosen because they represent the largest ethnic population (i.e. non-White and non-Aboriginal), accounting for over 25% of the ethnic population [26].

## Methods

### Study population

This cross-sectional survey consisted of Chinese and White adults, living in the city of Calgary, Alberta, Canada from February to June 2005. The city is culturally diverse and individuals of Chinese descent make up the largest ethnic group among the ethnic population comprising 5.5% of the city's 1 million population [27]. We screened the surnames listed in the Calgary telephone directory in 2004 using the validated Chinese surname list to form the Chinese sampling frame from which a random sample was taken [28]. After exclusion of screened Chinese names from the telephone directory, we randomly selected phone numbers from the remaining numbers for the White sample.

The randomly selected telephone numbers were called and only one respondent was interviewed in each household. If there was more than one eligible respondent in the household, the one with the latest birth date was selected for the interview. We surveyed respondents meeting the following criteria: 1) self-identification as either Chinese or White, 2) 18 years of age or older, 3) a resident of Canada for a minimum of one year, and 4) contact with a physician in Canada. We excluded people who could not speak English, Cantonese or Mandarin. For the White sample, ethnicity of respondents was determined through the question: "People living in Canada come from many different cultural and racial backgrounds. How would you describe your ethnic origin?" The choices of White, Chinese, and other were provided. For the Chinese sample, we asked persons who answered the telephone: "Is there anyone at your household whose ethnic background is Chinese?" We interviewed individuals who answered 'Yes" and met the inclusion criteria.

### Questionnaire development and translation

The questionnaire was first developed in English, including sociodemographic variables (gender, age, birthplace, length of stay in Canada, education level, language mainly spoken at home, marital status, and household income), presence of 15 chronic medical conditions (i.e. allergies, arthritis, back pain, cancer, depression, diabetes, headaches, heart disease, high blood pressure, kidney disease, liver disease, lung disease, neurological disorder including stroke, sleeping problems, and stomach diseases), past contact with physicians and perceptions of physicians.

For those who had visited a GP or specialist in Canada, we asked: "1) How satisfied were you with the care you received from the doctor? 2) Did you feel that the doctor had adequate experience to treat your condition? 3) Did you feel comfortable asking the doctor questions? 4) Did you feel the doctor spent enough time with you? 5) Did you understand what the doctor said to you? 6) Did you feel the doctor listened carefully to you? 7) Do you trust the doctor so much that you always try to follow his/her advice?" The five-point Likert scale was used to measure level of satisfaction (very satisfied, satisfied, neither, dissatisfied and very dissatisfied). A four-point scale (yes definitely, yes to some extent, no and unsure) was employed to measure patients' compliance with physician advice, comfort level asking physician questions, opinion on physician's clinical experience, adequacy of time spent, clarity, and attentiveness.

The English questionnaire was forwardly and backwardly translated into Chinese. A different translator did each of these translations. An investigator fluent in both English and Chinese reviewed the back-translated English version. The questionnaire was tested among 11 Chinese and White adults using face-to-face interviews. The English and Chinese questionnaires were further revised based on comments and suggestion made by the test groups.

### Data collection

Telephone interviewers were trained and practiced interviewing each other before the survey. Each of the randomly selected telephone numbers was called at different times of the day (i.e. evenings during weekdays and day time during weekends) up to 10 times. The Chinese survey was conducted in the respondent's preferred language by trained Chinese interviewers who were bi- or trilingual, speaking English, and Cantonese and/or Mandarin. The survey was conducted in English in the White sample. The University of Calgary Conjoint Health Research Ethics Board approved the survey and study methods.

### Data analysis

Characteristics of the study population were described by comparing proportions within the two groups. Chi-square was employed to test the statistical significance of differences in study variables between the Chinese and White groups. Dichotomous dependent variables were constructed by letting the response 'very satisfied or satisfied" or "Yes, definitely" equal one and zero otherwise. We regressed the binary dependent variables onto demographic and health condition variables using logistic regressions. The survey was designed to achieve several objectives. Hence, no power calculations were conducted to determine the sample size for this particular study. However, the sample size of 711 for each ethic population was sufficient to detect the difference between two proportions (e.g. 70% and 63%) with statistical power of 0.80 at P value = 0.05.

## Results

### Satisfaction, compliance and perceptions of physicians

Of the 1727 Chinese and 1948 Whites contacted by phone, 850 (49.2%) Chinese and 805 (41.3%) Whites responded the survey. After exclusion of respondents without contact with either GPs or specialists, 746 Chinese and 711 Whites were analyzed in the GP visit group and 485 Chinese and 637 Whites were analyzed in the specialist visit group.

Table 1 describes the characteristics of the White and Chinese respondents in the GP and specialist visit groups. Compared with Whites surveyed, greater proportions of Chinese were younger than 65 years old, male, had university or higher education, and were married. Also, the Chinese surveyed had lower incomes on average than the Whites surveyed. Small proportions of Chinese spoke English at home (10.7%) or were born in Canada (8.8%). Additionally, Chinese tended to have fewer chronic health problems than Whites.

**Table 1:** Characteristics of Chinese and White Survey Respondents who Visited General Practitioners or Specialists

	**General Practitioner Visits**			**Specialist Visits**		
Variables	Chinese N (% of 746)	White N (% of 711)	P-Value	Chinese N (% of 485)	White N (% of 637)	P-Value
Age						
18–34	188 (25.2)	179 (25.2)	< 0.001	100 (20.6)	145 (22.8)	0.036
35–64	497 (66.6)	428 (60.2)		335 (69.1)	398 (62.5)	
≥65	61 (8.2)	104 (14.6)		50 (10.3)	94 (14.8)	
						
Gender						
Male	355 (47.6)	251 (35.3)	< 0.001	204 (42.1)	225 (35.3)	0.021
Female	391 (52.4)	460 (64.7)		281 (57.9)	412 (64.7)	
						
Education						
Less than senior High	91 (12.2)	46 (6.5)	< 0.001	68 (14.0)	39 (6.1)	< 0.001
Senior High	127 (17.0)	187 (26.3)		92 (19.0)	166 (26.1)	
Technical/Professional College	147 (19.7)	232 (32.6)		103 (21.2)	204 (32.0)	
University or Higher	381 (51.1)	246 (34.6)		222 (45.8)	228 (35.8)	
						
Marital Status						
Married/common-law	585 (78.4)	426 (59.9)	< 0.001	388 (80.0)	385 (60.4)	< 0.001
Never married	118 (15.8)	170 (23.9)		63 (13.0)	152 (23.9)	
Divorced or Separated	43 (5.8)	115 (16.2)		34 (7.0)	100 (15.7)	
						
Income						
≤30,000	204 (27.3)	86 (12.1)	< 0.001	124 (25.6)	74 (11.6)	< 0.001
30,001–50,000	165 (22.1)	123 (17.3)		97 (20.0)	116 (18.2)	
50,001–70,000	112 (15.0)	101 (14.2)		87 (17.9)	88 (13.8)	
> 70,000	181 (24.3)	282 (39.7)		124 (25.6)	260 (40.8)	
Missing	84 (11.3)	119 (16.7)		53 (10.9)	99 (15.5)	
						
Language Spoken at Home						
English	80 (10.7)	688 (96.8)	< 0.001	64 (13.2)	616 (96.7)	< 0.001
Chinese	540 (72.4)	0 (0.0)		335 (69.1)	0 (0.0)	
Both English and Chinese	123 (16.5)	0 (0.0)		84 (17.3)	0 (0.0)	
Other	3 (0.4)	23 (3.2)		2 (0.4)	21 (3.3)	
						
Birthplace						
Canada	66 (8.8)	607 (85.4)	< 0.001	44 (9.1)	545 (85.6)	< 0.001
Other Countries	680 (91.2)	104 (14.6)		441 (90.9)	92 (14.4)	
						
Length of Stay in Canada Among						
Those Born Outside of Canada						
1 – 4 years	269 (39.6)	7 (6.7)		143 (32.4)	4 (4.3)	
5 – 9 years	135 (19.9)	15 (14.4)		86 (19.5)	13 (14.1)	
≥10 years	276 (40.6)	82 (78.8)		212 (48.1)	75 (81.5)	
						
Number of Conditions (out of 15)						
Zero conditions	337 (45.2)	141 (19.8)	< 0.001	194 (40.0)	112 (17.6)	< 0.001
1 condition	213 28.6)	164 (23.1)		140 (28.9)	149 (23.4)	
2 conditions	117 (15.7)	142 (20.0)		82 (16.9)	125 (19.6)	
3 conditions	43 (5.8)	106 (14.9)		37 (7.6)	94 (14.8)	
≥4 conditions	36 (4.8)	158 (22.2)		32 (6.6)	157 (24.6)	

Table 2 shows patients' reported satisfaction, compliance and perceptions of physicians in the two ethnic groups. Compared with Whites, a lower proportion of Chinese reported very satisfied or satisfied with GP (73.7% vs. 92.8%) and specialist care (75.5% vs. 85.6%) and the differences between these two groups remained after adjustment for demographic variables and chronic conditions (risk adjusted odds ratio (OR): 0.70, 95% confidence interval (95%CI): 0.42–1.18 for GP visit group and OR: 0.77, 95%CI: 0.48–1.23 for specialist visit group, see Table 3). A similar proportion of Chinese and Whites reported that they always followed physician's advice (59.4% vs. 59.6% for the GP visit group and 67.2% vs. 62.8% for the specialist visit group). In both GP and specialist visit groups, significantly fewer Chinese than Whites reported that physicians had adequate experience (44.2% vs. 82.4% for the GP visit group, risk adjusted OR: 0.40, 95%CI: 0.27–0.60 and 58.8% vs. 84.5% for the specialist visit group, risk adjusted OR: 0.49, 95%CI: 0.31–0.77). For the GP visit group, Chinese were less likely than Whites to report that physicians spent enough time with them (33.8% vs. 73.7%), that they felt comfortable asking questions (73.6% vs. 89.6%), and that they thought the physician listened to them carefully (63.4% vs. 82.3%). This difference remained after adjustment for demographic variables and chronic conditions and all the adjusted ORs were statistically significant at the 95% confidence level.

**Table 2:** Disaggregated responses among Chinese and Whites who have visited General Practitioners or Specialists

	General Practitioner Visits			Specialist Visits		
Variables	Chinese N (% of 746)	White N (% of 711)	P-Value	Chinese N (% of 485)	White N (% of 637)	P-Value
Satisfied with physician care						
Very Satisfied	158 (21.2)	420 (59.1)	0.0001	140 (28.9)	364 (57.1)	0.0001
Satisfied	392 (52.5)	240 (33.8)		226 (46.6)	181 (28.4)	
Neither	153 (20.5)	30 (4.2)		89 (18.4)	33 (5.2)	
Dissatisfied	33 (4.4)	17 (2.4)		20 (4.1)	25 (3.9)	
Very Dissatisfied	5 (0.7)	2 (0.3)		7 (1.4)	23 (3.6)	
Unsure	5 (0.7)	2 (0.3)		3 (0.6)	11 (1.7)	
						
Definitely trust physician enough to always follow physician advice						
Yes Definitely	443 (59.4)	424 (59.6)	0.606	326 (67.2)	400 (62.8)	0.003
Yes, to some extent	267 (35.8)	242 (34)		131 (27)	158 (24.8)	
No	29 (3.9)	37 (5.2)		21 (4.3)	62 (9.7)	
Unsure	7 (0.9)	8 (1.1)		7 (1.4)	17 (2.7)	
						
Definitely felt physician had adequate experience						
Yes Definitely	330 (44.2)	586 (82.4)	0.0001	285 (58.8)	538 (84.5)	0.0001
Yes, to some extent	361 (48.4)	103 (14.5)		153 (31.5)	67 (10.5)	
No	41 (5.5)	12 (1.7)		28 (5.8)	19 (3)	
Unsure	14 (1.9)	10 (1.4)		19 (3.9)	13 (2)	
						
Definitely felt enough time was spent with physician						
Yes Definitely	252 (33.8)	524 (73.7)	0.0001	212 (43.7)	404 (63.4)	0.0001
Yes, to some extent	335 (44.9)	132 (18.6)		187 (38.6)	155 (24.3)	
No	149 (20)	52 (7.3)		81 (16.7)	68 (10.7)	
Unsure	10 (1.3)	3 (0.4)		5 (1)	10 (1.6)	
						
Definitely felt comfortable asking physician questons						
Yes Definitely	549 (73.6)	637 (89.6)	0.0001	343 (70.7)	494 (77.6)	0.001
Yes, to some extent	173 (23.2)	61 (8.6)		108 (22.3)	85 (13.3)	
No	19 (2.5)	13 (1.8)		26 (5.4)	49 (7.7)	
Unsure	4 (0.7)	0 (0)		8 (1.6)	9 (1.4)	
						
Definitely understood physician explanation and advice						
Yes Definitely	567 (76)	667 (93.8)	0.0001	278 (57.3)	516 (81)	0.0001
Yes, to some extent	156 (20.9)	41 (5.8)		157 (32.4)	99 (15.5)	
No	18 (2.4)	2 (0.3)		49 (10.1)	16 (2.5)	
Unsure	5 (0.7)	1 (0.1)		1 (0.2)	6 (0.9)	
						
Definitely felt physician listened carefully						
Yes Definitely	473 (63.4)	585 (82.3)	0.0001	289 (59.6)	456 (71.6)	0.0001
Yes, to some extent	238 (31.9)	112 (15.8)		167 (34.4)	121 (19)	
No	31 (4.2)	14 (2)		21 (4.3)	50 (7.8)	
Unsure	4 (0.5)	0 (0)		8 (1.6)	10 (1.6)	

**Table 3:** Satisfaction, Compliance, and Perceptions of Physicians among Chinese and Whites who have Visited General Practitioners or Specialists

	General Practitioner Visits				Specialist Visits			
Variables	Chinese N (% of 746)	White N (% of 711)	P-Value*	Adjusted OR (95% CI)^#^Chinese vs. White	Chinese N (% of 485)	White N (% of 637)	P-Value*	Adjusted OR (95% CI)^#^Chinese vs. White
Satisfied with physician care	550 (73.7)	660 (92.8)	< 0.001	0.70 (0.42–1.18)	366 (75.5)	545 (85.6)	< 0.001	0.77 (0.48–1.23)
Definitely trust physician enough to always follow physician advice	443 (59.4)	424 (59.6)	0.922	1.02 (0.69–1.50)	326 (67.2)	400 (62.8)	0.125	1.26 (0.82–1.94)
Definitely felt physician had adequate experience	330 (44.2)	586 (82.4)	< 0.001	0.40 (0.27–0.60)	285 (58.8)	538 (84.5)	< 0.001	0.49 (0.31–0.77)
Definitely felt enough time was spent with physician	252 (33.8)	524 (73.7)	< 0.001	0.38 (0.26–0.56)	212 (43.7)	404 (63.4)	< 0.001	0.66 (0.44–1.00)
Definitely felt comfortable asking physician questions	549 (73.6)	637 (89.6)	< 0.001	0.53 (0.34–0.82)	343 (70.7)	494 (77.6)	0.009	1.02 (0.65–1.60)
Definitely understood physician explanations and advice	567 (76.0)	667 (93.8)	< 0.001	0.67 (0.38–1.19)	278 (57.3)	516 (81.0)	< 0.001	0.74 (0.45–1.20)
Definitely felt physician Listened carefully	473 (63.4)	585 (82.3)	< 0.001	0.53 (0.35–0.82)	289 (59.6)	456 (71.6)	< 0.001	0.80 (0.52–1.22)

Note:

* P-value is crude without risk adjustment

^# ^OR: Odds Ratio

95%CI: 95% Confidence Interval

Age, gender, education, marital status, income, language, birthplace, and number of chronic conditions were adjusted for calculation of adjusted odds ratio

### Factors associated with satisfaction, compliance and perceptions of physicians among Chinese respondents

We restricted our analyses to Chinese only in order to assess factors associated with their reported satisfaction and compliance (see Table 4). After adjustment for sociodemographics and chronic health conditions, language spoken at home and birthplace were statistically significantly associated with their reported satisfaction, compliance and perceptions of physicians.

**Table 4:** Factors Associated with Satisfaction, Compliance, and Perceptions of Physicians among Chinese who Visited General Practitioners or Specialists

Variables	Satisfaction OR (95%CI)*	Compliance OR (95%CI)*	Experience OR (95% CI)*	Time OR (95%CI)*	Questions OR (95%CI)*	Clarity OR (95%CI)*	Attentiveness OR (95%CI)*
Chinese Patients who Visited General Practitioners (n = 746)							

Language							
English	1.0	1.0	1.0	1.0	1.0	1.0	1.0
Chinese or Other	0.5 (0.3–0.9)	1.2 (0.8–1.7)	0.7 (0.4–1.0)	1.0 (0.7–1.6)	0.8 (0.5–1.3)	0.7 (0.4–1.2)	0.9 (0.6–1.4)
							
Length of Stay in Canada							
Born in Canada	1.0	1.0	1.0	1.0	1.0	1.0	1.0
1 – 9 years	0.2 (0.1–0.7)	1.1 (0.5–2.1)	0.3 (0.2–0.7)	0.2 (0.1–0.5)	0.4 (0.2–1.0)	0.3 (0.1–0.9)	0.5 (0.3–1.1)
10 or more years	0.5 (0.1–1.7)	1.4 (0.7–2.7)	0.8 (0.4–1.6)	0.6 (0.3–1.1)	0.7 (0.3–1.6)	0.7 (0.2–1.9)	0.7 (0.4–1.5)

Chinese Patients who Visited Specialists (n = 485)							

Language							
English	1.0	1.0	1.0	1.0	1.0	1.0	1.0
Chinese or Other	0.7 (0.4–1.2)	1.5 (0.9–2.5)	1.0 (0.6–1.7)	0.6 (0.4–1.0)	0.6 (0.4–1.1)	0.4 (0.2–0.7)	0.8 (0.5–1.3)
							
Length of Stay in Canada							
Born in Canada	1.0	1.0	1.0	1.0	1.0	1.0	1.0
1 – 9 years	0.5 (0.1–1.6)	0.3 (0.1–0.8)	0.2 (0.1–0.6)	0.4 (0.2–1.0)	0.7 (0.2–1.9)	1.0 (0.4–2.6)	0.6 (0.2–1.5)
10 or more years	0.5 (0.2–1.8)	0.5 (0.2–1.3)	0.3 (0.1–0.9)	0.5 (0.2–1.2)	0.7 (0.3–2.0)	1.4 (0.5–3.7)	0.5 (0.2–1.3)

Note: *Odds ratios (95% Confidence Intervals) were calculated after adjustment for age, sex, education, marital status, income, medical conditions, language and birthplace

Questions asked in the Survey:

Experience: Did you feel that the doctor had adequate experience to treat your condition?

Questions: Did you feel comfortable asking the doctor questions?

Time: Did you feel the doctor spent enough time with you?

Clarity: Did you understand what the doctor said to you?

Attentiveness: Did you feel the doctor listened carefully to you?

Compliance: Did you trust the doctor so much that you always try to follow his/her advice?

Satisfaction: How satisfied were you with the care you received from the doctor?

Non-English speaking Chinese and recent arrivals in Canada were less likely to be satisfied with GPs than Chinese born in Canada (risk adjusted OR: 0.5, 95%CI: 0.3–0.9, 0.2 and 0.1–0.7, respectively). Recent immigrants also rated GPs significantly lower than Canadian-born Chinese in terms of perceived clinical experience (risk adjusted OR: 0.3, 95% CI: 0.2–0.7), clarity of physicians' communication (risk adjusted OR: 0.3, 95% CI: 0.1–0.9), and adequate time spent (risk adjusted OR: 0.2, 95% CI: 0.1–0.5).

Recent immigrants were less likely to report following a specialist's advice compared with Canadian-born Chinese (OR: 0.3, 95% CI: 0.1–0.8). Immigrants were also more likely to believe their specialist lacked clinical experience compared with Canadian-born Chinese (OR: 0.2, 95% CI: 0.1–0.6 for recent immigrants and OR: 0.3, 95% CI: 0.1–0.9 for those residing in Canada for 10 years or longer).

## Discussion

We surveyed Chinese and White Canadians to examine their reported satisfaction, compliance and perceptions of physicians. We found that Chinese Canadians' physician satisfaction and perceptions differed from White Canadians. These findings were consistent with lower satisfaction ratings for physicians found among ethnic populations compared with Whites in the United Status, United Kingdoms and Australia [13-21]. This reported difference might be attributed to the views from our sample of recently immigrated Chinese. Our sample of recent immigrants was mainly from China (i.e. mainland China and Hong Kong) where the health care system is different from Canada's. China, particularly China's mainland, does not have an established primary care (GP) system and patients mainly visit physicians at outpatient departments of hospitals without appointments. Patients or a third party pay for physician services. Therefore, physicians in China attempt to satisfy their patients by providing prescriptions and diagnostics based on patient requests [29,30]. Recent immigrants to Canada might maintain these same expectations in their interactions with Canadian physicians. However, in Canada, physicians are seen primarily by appointment and necessity determines the prescription of drugs and diagnostics. These cultural factors may also have played a role in our finding that recent Chinese immigrants were more likely than non-immigrants to report that physicians lacked clinical experiences and did not spend enough time with them.

Chinese Canadians that did not speak English reported less satisfaction than those who spoke the language. These findings suggest that clear communication between physicians and patients still plays a significant role in patient perceptions of physicians. Hence, this is an area that should be improved when providing healthcare services. Translation services or selecting physicians proficient in a patient's first language may have some value [31-34]. It is notable, however, that patients who did not speak English reported following physician advice to the same degree as those speaking English. Our findings suggest that language and communication are important factors for patient satisfaction but satisfaction is not necessarily correlated with patient compliance in the Chinese population.

Our study showed that a higher proportion of Chinese Canadians reported neutral levels of satisfaction (neither satisfied nor dissatisfied) and perception (yes to some extent) than White Canadians. This result might be related to Chinese cultural tendencies towards midpoint responses in measures associated with feelings or traits [35]. However, it is difficult to objectively quantify the effect of this cultural subjectivity on our measures without a population-based 'gold standard'. Hence, it should be emphasized that our study measured self-perceived rather than actual satisfaction and perceptions of physicians. Our results represented the combined effect of cultural subjectivity and expectations of physicians. We dichotomized our satisfaction and perception measurements to capture patients who were 'definitively' satisfied or had a 'definitive' perception towards physicians. Our findings demonstrated an existing gap between Chinese and White Canada when self-perceived 'satisfaction' was used as a measure of the healthcare system or physician performance.

A limitation of this study is recall bias because our findings relied on participant recollection of their recent physician experiences. Second, the survey was conducted only in Calgary and involved only one of the many Canadian ethnic minorities. It is uncertain if these findings are generalizable to other ethnic populations. Third, the survey questions were designed for this study only and their validity was not assessed. Fourth, our response rate was not optimal and we could not quantify the differential bias between respondents and non-respondents in satisfaction and compliance. If Chinese respondents, for example, were more likely to have positive perceptions about their physician relative to Chinese non-respondents, we would produce over-estimates of satisfactions. Lastly, we did not ask respondents' for their physicians' ethnicity or comfort with the language spoken with the physician. Therefore, the impact of ethnic concordance between the patient and physician on satisfaction could not be assessed.

## Conclusion

Chinese reported less satisfaction with physicians and perceived physicians slightly more negatively than Whites. Chinese with limited English and short length of stay in Canada reported less satisfaction than Canadian born Chinese. Physicians should strive to better fill the needs of their patients by paying particular attention to clarity and ensuring patient understanding. Possible tools for achieving this goal include the use of educational aids, language translation services and communication-specific physician training, specifically for recent immigrants and those with limited English language capacity. Physicians should also be aware that new immigrants may have different healthcare expectations Physicians should be sensitive to these possible cultural differences.

## Competing interests

The author(s) declare that they have no competing interests.

## Authors' contributions

RL participated in the statistical analysis of the paper and drafted the manuscript. LS performed the statistical analysis and critically reviewed the paper. HQ conceived of the study, participated in its design and coordination and helped to draft the manuscript. All authors read and approved the final manuscript.

## Pre-publication history

The pre-publication history for this paper can be accessed here:


